# Peripheral Cemento-Ossifying Fibroma: Case Series Literature Review

**DOI:** 10.1155/2013/930870

**Published:** 2013-01-14

**Authors:** Kiran Kumar Ganji, ArunKumar Bhimashankar Chakki, Sharanbasappa Chandrashekar Nagaral, Esha Verma

**Affiliations:** ^1^Department of Periodontics, Sharad Pawar Dental College, Datta Meghe Institute of Medical Sciences (Deemed University), Nagpur, India; ^2^Department of Oral and Maxillofacial Pathology, Guru Gobind Singh College of Dental Sciences, Devi Ahilya University, Burhanpur, Indore, India; ^3^Department of Prosthodontics, Al-badar Dental College and Hospital, Near PDA Engineering College, Rajiv Gandhi University of Health Sciences, Gulbarga, Bangalore, India; ^4^Department of Periodontics, College of Dental Sciences & Hospital, Devi Ahilya University, Rau, Indore, India

## Abstract

The concept of fibroosseous lesions of bone has evolved over the last several decades and now includes two major entities: fibrous dysplasia and ossifying fibroma. Peripheral cemento-ossifying fibroma is a relatively rare tumour classified between fibroosseous lesions. It predominantly affects adolescents and young adults, with peak prevalence between 10 and 19 yrs. The cemento-ossifying fibroma is a central neoplasm of bone as well as periodontium which has caused considerable controversy because of confusion regarding terminology and the criteria for its diagnosis. The cemento-ossifying fibroma is odontogenic in origin, whereas ossifying fibroma is of bony origin. Lesions histologically similar to peripheral ossifying fibroma have been given various names in existing literature. Therefore, we present and discuss in this paper a series of cases of peripheral cemento-ossifying fibroma emphasizing the differential diagnosis.

## 1. Introduction

Benign fibroosseous lesions of the jaws present problems in diagnosis and classification. The 1992 WHO classification groups under a single designation (cemento-ossifying fibroma) two histologic types (cementifying fibroma and ossifying fibroma) that may be clinically and radiographically undistinguishable [1]. Cemento-ossifying fibroma is a relative rare lesion considered as an osteogenic tumor (nonodontogenic) with variable expressiveness. It is defined as a well-demarcated and occasionally encapsulated lesion consisting of fibrous tissue containing variable amounts of mineralized material resembling bone (ossifying fibroma), cementum (cementifying fibroma), or both [2, 3].

Peripheral cemento-ossifying fibroma (PCOF) accounts for 3.1% of all oral tumors [4] and for 9.6% of gingival lesions [5]. The pathogenesis of this tumor is uncertain. Due to their clinical and histopathological similarities, some PCOFs are believed to develop fibrous maturation and subsequent calcification. PCOF is frequently associated with irritant agents such as calculus, bacterial plaque, orthodontic appliances, ill adapted crowns, and irregular restorations. The mineralized product probably originates from periosteal cells or from the periodontal ligament [6]. PCOF affects both genders, but a higher predilection for females has been reported in the literature [4]. With respect to race, there is a predominance in Whites (71%) compared to Blacks (36%) [7]. It may occur at any range, but exhibits a peak incidence between the second [8] and third decades [7]. However, Neville et al. [9] say that it predominantly affects adolescents and young adults, with a peak prevalence between 10 and 19 years.

Clinically, PCOF manifests as a pediculate or sessile nodular mass, which usually originates in the interdental papilla. Its color is similar to that of the mucosa unless the lesion is ulcerated. Most tumors measure less than 2 cm in diameter, although lesions larger than 10 cm are occasionally observed. About 60% of the tumors occur in the maxilla and more than 50% of all cases affect the region of the incisors and canines. A potential of tooth migration PCOF has been reported [6]. Hence, the purpose of this paper is to present a series of cases of PCOF lesions and emphasize the importance of discussion of the reasonable differential diagnosis with the patient.

## 2. Case Description

**Figure 1:**
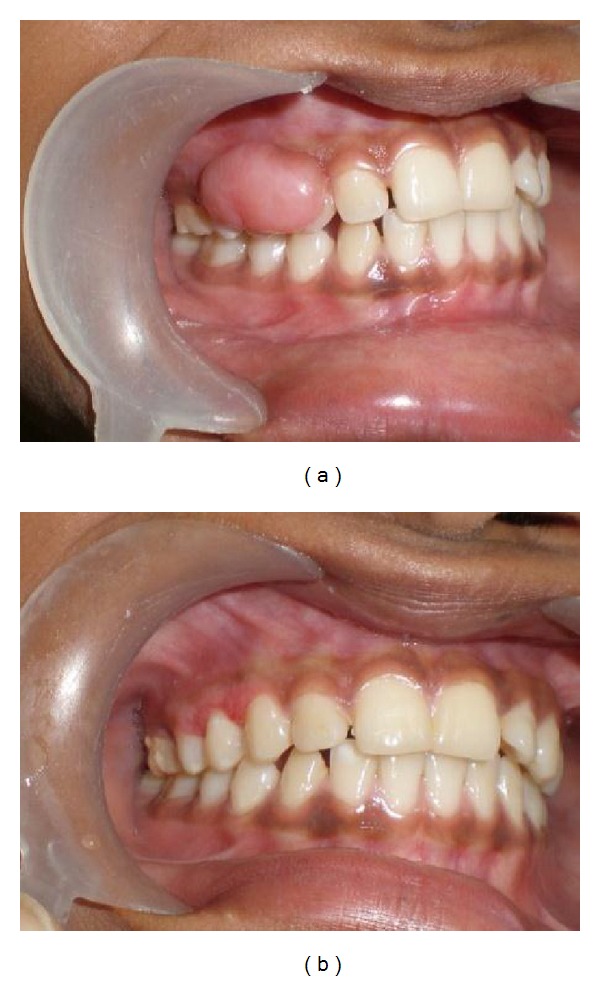
(a) Clinical view of lesion. (b) Postoperative view.

*Case  1*. A 21-year-old male reported to College of Dental Sciences & Hospital, Rau, Indore, India, with his slow growing, painless growth that had been present facial in upper right premolar to molar region. Lesion started as a small papule approximately 1 year earlier. According to the patient, there was no bleeding and pain except difficulty in mastication. Examination revealed approximately 2 × 1.5 cm pedunculated nontender, firm, pinkish red growth present on the buccal gingival in relation to maxillary right canine to 1st molar. The lesion extended up to the level of occlusal plane and revealed indentations made by the occluding mandibular premolar. The surface of the occlusal plane was pinkish red in color (Figure 1).

**Figure 2:**
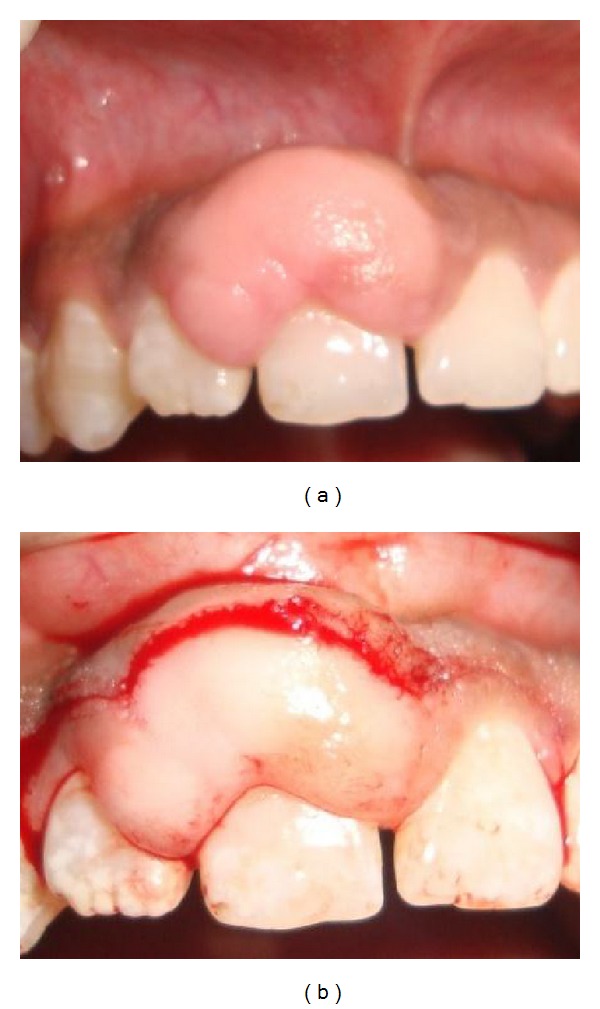


*Case  2*. A healthy 25- year-old male reported to College of Dental Sciences & Hospital, Rau, Indore, India, with a lump in his back teeth. According to the patient, the reddish purple lump has been present for approximately 6 months and the lump was interfering with his bite and felt uncomfortable. Occasionally bleeding occurred when he brushed his teeth. Clinical examination revealed erythematous interdental papilla in relation to maxillary central incisors 11,12 visible from facial aspect with no evidence of lesion palatally. The lesion appeared exophytic and nodular with irregular surface. It measured approximately 10 mm laterally and 8 mm in anterior-posterior direction and 6 mm thick. It was slightly pedunculated with what appeared to be a broad-based attachment. The lesion was neither fluctuant nor did it blanch on pressure, but had a rubbery consistency. It was tender (Figure 2). 

**Figure 3:**
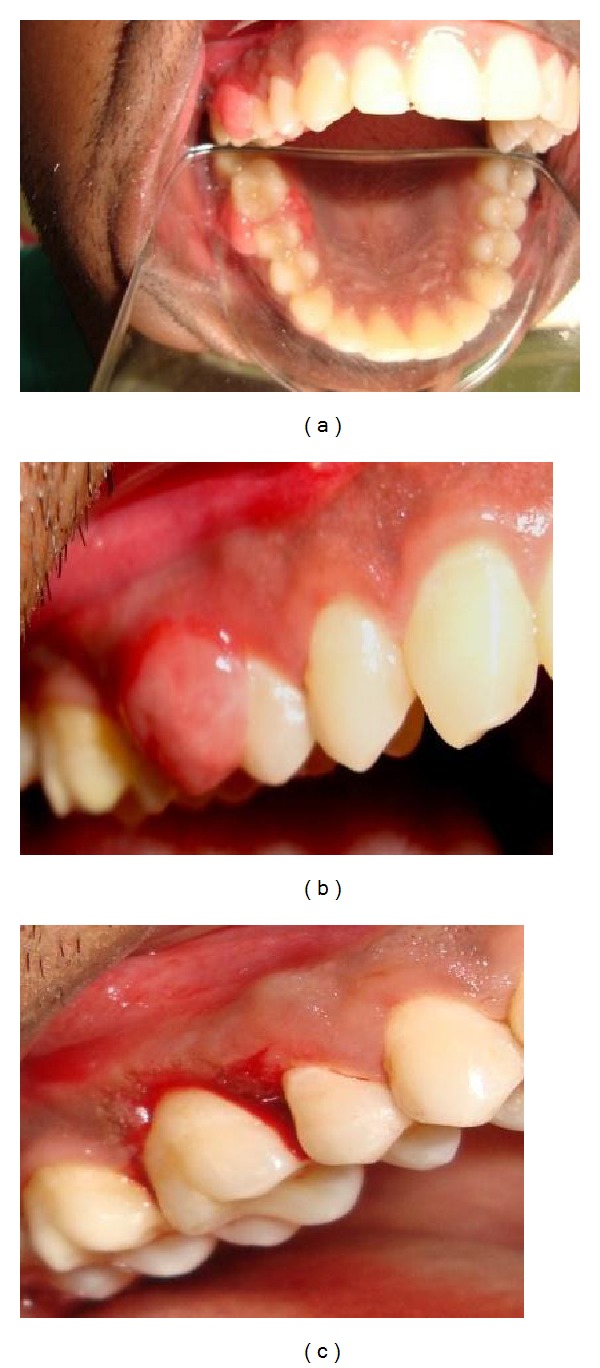
(a) Clinical view. (b) Clinical view. (c)View after excision.

*Case  3*. A 26-year-old male patient was referred by his general dental practitioner for gingival swelling in relation 15 to 16 region. The past dental history revealed presence of swelling since last one and half year duration. On examination, the associated soft tissue was slightly swollen but there was no ulceration, on palpation the swelling was soft rubbery in consistency, but no tenderness. The lesion was well demarcated and pedunculated measuring approximately 1.5 × 2 cm (Figure 3).

**Figure 4:**
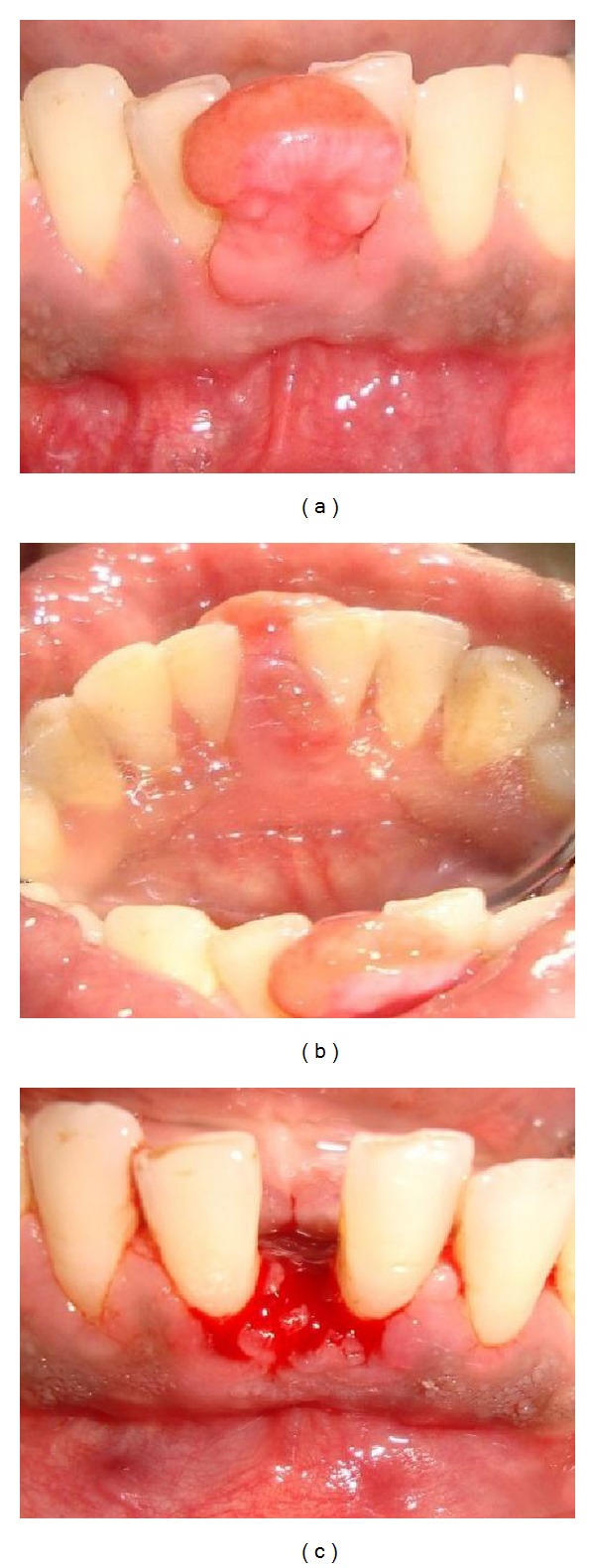
Clinical view.

*Case  4*. A 31-year-old male reported to the College of Dental Sciences & Hospital, Rau, Indore, India complaining of inability to chew food since 6 to 8 months. The patient was apparently asymptomatic 18 months back when he developed a small swelling in the mandibular anterior labial region in 31, 41 which gradually increased in size. On examination, a uniform rounded swelling was present in mandibular anterior region due to which the patient could not chew the food. The size of the lesion was 2.5 × 2 cm and the shape was ovoid. The overlying mucosa was pinkish in color and firm in consistency. The texture was smooth. There was no compressibility or depressibility (Figure 4).

## 3. Diagnosis 

Differential diagnosis consisted of irritational fibroma, pyogenic granuloma, peripheral giant cell granuloma, peripheral cemento-ossifying fibroma.

**Figure 5:**
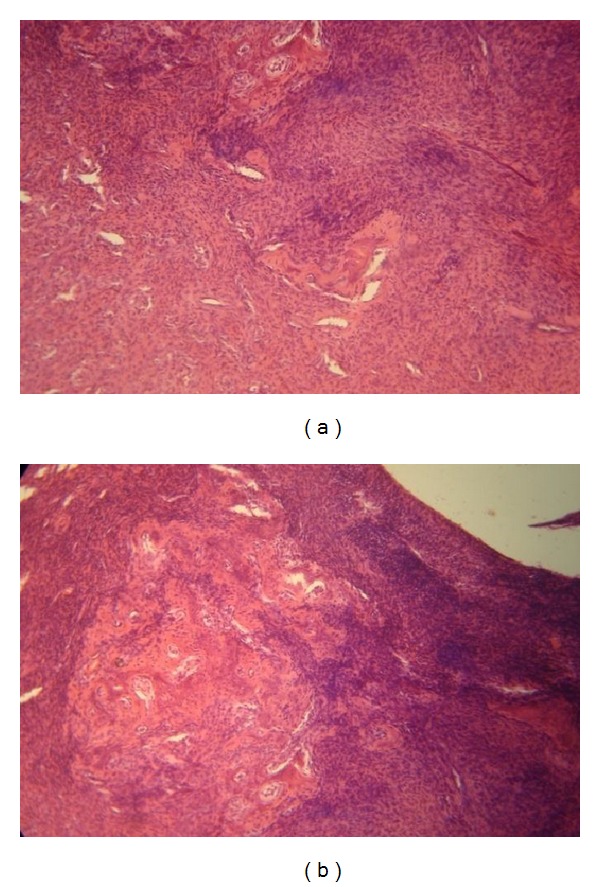
Histologic view of the lesion under 20x magnification.

A confirmatory diagnosis of peripheral cemento-ossifying fibroma is made by histopathologic evaluation of biopsy specimens. The following features were observed during microscopic examination:

1. ulcerated stratified squamous surface epithelium;
2. benign fibrous connective tissue with varying numbers of fibroblasts;
3. sparse-to-profuse endothelial proliferation;
4. mineralized material consisting of mature, lamellar or woven osteoid, cementum like material, or dystrophic calcifications;
5. acute or chronic inflammatory cells in lesion (Figure 5).

## 4. Discussion

Peripheral ossifying fibroma is thought to be either reactive or neoplastic in nature. Considerable confusion has prevailed in the nomenclature of peripheral ossifying fibroma with various synonyms being used, such as peripheral cementifying fibroma, ossifying fibroepithelial polyp, peripheral fibroma with osteogenesis, peripheral fibroma with cementogenesis, peripheral fibroma with calcification, calcifying or ossifying fibroma epulis, and calcifying fibroblastic granuloma [10]. Ossifying fibromas elaborate bone, cementum and spheroidal calcifications, which has given rise to various terms for these benign fibroosseous neoplasms. When bone predominates, “ossifying” is the appellation, while the term “cementifying” has been assigned when curvilinear trabeculae or spheroidal calcifications are encountered [11]. When bone and cementum-like tissues are observed, the lesions have been referred to as cemento-ossifying fibroma [11]. Cementifying fibromas may be clinically and radiographically impossible to separate from ossifying fibromas [12]. An attempt has been made by Endo et al. to distinguish cementifying fibroma from ossifying fibromas and fibrous dysplasias by using immunohistochemical analysis for keratin sulfate and chondrotoin-4 sulfate in which the cementifying fibromas showed significant immunoreactivity for keratan sulfate and ossifying fibromas, and fibrous dysplasias showed intensive immunostaining for chondroitin-4-sulfate [12]. 

The term cemento-ossifying has been referred to as outdated and scientifically inaccurate [13] because the clinical presentation and histopathology of cemento-ossifying fibroma are the same in areas where there is no cementum, such as the skull, femur, and tibia. These are all ossifying fibromas; those that happen to occur in the jaws should not be termed cement ossifying fibromas merely because of the presence of teeth. Moreover, there is no histologic or biochemical difference between cementum and bone. Cemento-ossifying fibroma is the term given mainly due to presence of dysmorphic round basophilic bone particles within ossifying fibroma, which have arbitrarily been called cementicles. However, these so-called cementicles are not from cementum but instead represent a dysmorphic product of this tumour analogous to the keratin pearls, which are a dysmorphic product of squamous cell carcinoma [13]. 

Though the etiopathogenesis of peripheral ossifying fibroma is uncertain, an origin from cells of periodontal ligament has been suggested [10]. The reasons for considering periodontal ligament origin for peripheral ossifying fibroma include exclusive occurrence of peripheral ossifying fibroma in the gingiva (interdental papilla), the proximity of gingiva to the periodontal ligament, and the presence of oxytalan fibres within the mineralized matrix of some lesions [10]. Excessive proliferation of mature fibrous connective tissue is a response to gingival injury, gingival irritation, subgingival calculus, or a foreign body in the gingival sulcus. Chronic irritation of the periosteal and periodontal membrane causes metaplasia of the connective tissue and resultant initiation of formation of bone or dystrophic calcification. It has been suggested that the lesion may be caused by fibrosis of the granulation tissue [14]. 

Lesions involving the gingival soft tissues are rare compared to the lesions appearing within bone [12]. Mesquita RA found higher numbers of argyrophilic nucleolar organizer regions (AgNORs) and proliferating cell nuclear antigen- (PCNA-) positive cells in ossifying fibroma than in peripheral ossifying fibroma, indicating higher proliferative activity in ossifying fibroma [15]. X-ray diffraction analysis indicated that the mineral phase of both central and peripheral tissues consists of apatite crystals and that the crystallinity of the apatites might improve progressively with the development of the lesion, possibly to the same degree as that of bone apatite [16]. Peripheral ossifying fibroma tends to occur in the 2nd and 3rd decades of life, with peak prevalence between the ages of 10 and 19. 

Eversole and Rovin [17] stated the similar sex and site predilection of pyogenic granuloma. Gardner [18] stated that peripheral ossifying fibroma, cellular connective tissue is so characteristic that a histologic diagnosis can be made with confidence, regardless of the presence or absence of calcification. Buchner and Hansen [19] hypothesized that early POF presents as ulcerated nodules with little calcification, allowing easy misdiagnosis as a pyogenic granuloma. Although it is also important to maintain a high index of suspicion, discussion with family members should be tactful to prevent undue distress during the waiting period between differential diagnosis and definitive histopathologic diagnosis. Because the clinical appearance of these various lesions can be remarkably similar, classification is based on their distinct histologic differences. The POF must be differentiated from the peripheral odontogenic fibroma (PODF) described by the World Health Organization [18, 19]. Histologically, the PODF has been defined as a fibroblastic neoplasm containing odontogenic epithelium [20]. Despite a preponderance of the literature supporting differentiation, some authors continue to argue that the POF (or peripheral cemento-ossifying fibroma) is the peripheral counterpart of the central cemento-ossifying fibroma [21]. The POF, as discovered in this case, is a focal, reactive, nonneoplastic tumour-like growth of soft tissue often arising from the interdental papilla [19]. It is a fairly common lesion, comprising nearly 3% of oral lesions biopsied in 1 study 1 approximately 1%-2% in other studies [21]. In 1993, S. Das and A. Das [8] obtained similar results, with 1.6% POFs among 2,370 intraoral biopsies. 

POFs are believed to arise from gingival fibers of the periodontal ligament as hyperplastic growth of tissue that is unique to the gingival mucosa [17, 18]. This hypothesis is based on the fact that POFs arise exclusively on the gingiva, the subsequent proximity of the gingiva to the periodontal ligament, and the inverse correlation between age distribution of patients presenting with POF and the number of missing teeth with associated periodontal ligament [20]. The POF lesion is generally small and does not require imaging beyond radiographs [18]. Treatment consists of conservative surgical excision [20] and scaling of adjacent teeth [18]. Therefore, regular followup is required. Although peripheral ossifying fibroma is benign, reactive lesion, the recurrence rate is fairly high. Therefore, the patients are still under follow-up period.

## 5. Conclusions

POF is a slowly progressing lesion, the growth of which is generally limited. Many cases will progress for long periods before patients seek treatment because of the lack of symptoms associated with the lesion. A slowly growing pink soft-tissue nodule in the anterior maxilla of an adolescent should raise suspicion of a POF. Discussion of the differential diagnosis should be done tactfully to prevent unnecessary distress to the patient and family. Zhang and others [16] noted that cancer was included in the differential diagnosis in only 2% of cases. In the current case, the family experienced distress related to the suggestion of squamous cell carcinoma before referral for treatment and definitive diagnosis. Treatment consists of surgical excision, including the periosteum and scaling of adjacent teeth. Close postoperative followup is required because of the growth potential of incompletely removed lesions and the 8%–20% recurrence rate.
