# Erratum to: “Peripheral Cemento-Ossifying Fibroma: Case Series Literature Review”

**DOI:** 10.1155/2013/827247

**Published:** 2013-04-29

**Authors:** Esha Verma, ArunKumar Bhimashankar Chakki, Sharanbasappa Chandrashekar Nagaral, Kiran Kumar Ganji

**Affiliations:** ^1^Department of Periodontics, College of Dental Sciences & Hospital, Devi Ahilya University, Rau, Indore, India; ^2^Department of Oral and Maxillofacial Pathology, Guru Gobind Singh College of Dental Sciences, Devi Ahilya University, Burhanpur, Indore, India; ^3^Department of Prosthodontics, Al-badar Dental College and Hospital, Near PDA Engineering College, Rajiv Gandhi University of Health Sciences, Gulbarga, Bangalore, India; ^4^Department of Periodontics, Sharad Pawar Dental College, Datta Meghe Institute of Medical Sciences (Deemed University), Nagpur, India

In the paper titled “Peripheral Cemento-Ossifying Fibroma: Case Series Literature Review,” the order of the authors should be as shown here.

